# Bright compact ultrabroadband source by orthogonal laser-sustained plasma

**DOI:** 10.1038/s41377-024-01602-2

**Published:** 2024-09-26

**Authors:** Zhaojiang Shi, Shichao Yang, He Hu, Haodong Lei, Zhaohua Yang, Xia Yu

**Affiliations:** School of Instrumentation and Optoelectronic Engineering, Beihang University, Beijing, 100191 China

**Keywords:** Lasers, LEDs and light sources, Plasma physics

## Abstract

Laser-sustained plasma (LSP) source featuring high brightness and broadband spectral coverage is found to be powerful in various fields of scientific and industrial applications. However, the fundamental limit of low conversion efficiency constrains the system compactness and widespread applications of such broadband light sources. In this paper, we propose an innovative orthogonal LSP to break through the conversion efficiency limitation. Driven by the elevated conversion efficiency from absorbed laser power to ultraviolet (UV) emission, a compact broadband source (250–1650 nm) with UV spectral radiance exceeding 210 ${mW}/({{mm}}^{2}\,\cdot\, {sr}\,\cdot\, {nm})$ is achieved with >100 W pump laser. With the plot of a two-dimensional refractive index model, we report an important conceptual advance that the orthogonal design eliminates the influence of the negative lensing effect on laser power density. Experimental results unambiguously demonstrate that we achieve a bright compact UV-VIS-NIR source with negligible thermal loss and the highest conversion efficiency to our knowledge. Significant enhancement of 4 dB contrast-to-noise ratio (CNR) in spectral single-pixel imaging has been demonstrated using the proposed ultrabroadband source. By establishing the quantitative link between pumping optics design and plasma absorption, this work presents a compact broadband source that combines superior conversion efficiency and unprecedented brightness, which is essential to high-speed inspection and spectroscopy applications.

## Introduction

Bright ultraviolet-visible-near-infrared (UV-VIS-NIR) sources have attracted general interest in advanced inspection and spectroscopy applications, such as microcavity inspection^1^, advanced material characterization^2,3^, broadband detector calibration^4,5^, biomolecular dynamic monitoring^6,7^, near-field nanospectroscopy^8,9^, nanophotonics^10,11^, transient absorption spectroscopy^12^ and hyperspectral imaging^13,14^. High brightness is essential to determine the speed of defect inspection^15^, thus breaking through the bottleneck for improving the system throughput of advanced semiconductor processes. Similarly, high brightness UV-VIS-NIR source is critical to reduce the integration time required to obtain sufficient contrast-to-noise ratio (CNR), thus promoting the development of dynamic spectral imaging. Recently, synchrotron light sources^16,17^, supercontinuum light sources^18–21^, ultrabroadband light-emitting diodes^22,23^, constant-wave laser-driven white light emission^24^ have been developed to satisfy diverse demands. However, despite impressive progress, the average power of these UV-VIS-NIR sources falls short of what is required for the latest applications. Laser-sustained plasma (LSP) is a promising UV-VIS-NIR source with an average output power of tens or even hundreds of watts. The common implementation method for LSP is to generate the initial plasma in the high-pressured noble gas bulb (usually Xenon or Argon) by arc discharge. Subsequently, laser beams are focused on the plasma and provide the power for its sustenance instead of electric current. LSP has a temperature of more than 10,000 K and a luminous size of several hundred microns, resulting in high average output power, wide spectral range, and high brightness^25,26^. We provide the comparison between LSP sources and other broadband sources in the discussion section Table 1.

**Table 1:** Comparison of broadband sources

Light source	Wavelength range	Brightness *I*	Average output power *P* (mW)	Footprint *V* (m³)	Temporal coherence	Figure-of-merit (*FOM* = *∆λ IP/V*)
Supercontinuum light source^20^	340 nm–40 μm	12990 mW/mm²/sr/nm @350 nm	70 mW	~1 m³	Coherent pulse	~10^10^
Synchrotron light source^20^	8 pm–517 μm	730 mW/mm²/sr/nm @350 nm	~600 mW @350 nm	~3 × 10^5^ m^3^	Coherent pulse	~10^9^
Perovskite LED^23^	520–580 nm	161900 cd/m^2^	~0.4 mW	~10^−8^ m^3^	Incoherent CW	~10^9^
LSP source (this work)	250–1650 nm	267 mW/mm²/sr/nm @350 nm	>20 × 10^3^ mW	~0.02 m^3^	Incoherent CW	~10^11^

With the surpassed figure-of-merit (FOM), LSP is a promising broadband source for industrial applications. However, due to the negative lensing effect of plasma, the laser transmission path is deflected, and the typical conversion efficiency of LSP is capped at <10%. This low conversion efficiency results in bulk laser and extremely complex thermal management structure utilized for higher brightness LSP sources^27^, which limits their application from the perspective of compact system integration. Therefore, bright and compact LSP sources driven with <100 W laser power will generate significant interest in high-speed inspection and spectroscopy applications.

In this paper, we propose an innovative solution to break through the conversion efficiency limitation of LSP sources. Herein, the design of orthogonal lasers is demonstrated to suppress the influence of negative lens effect on laser power density. Precision tuning of the off-focal crossing point is to maximize the beam overlap and, hence, the plasma absorption. This innovation helps to establish the quantitative link between pumping optics design and plasma absorption. The overall conversion efficiency here is defined as the ratio of UV range (300–370 nm) emission power to the total laser power that pumps the plasma. When the laser power is 90 W, the spectral radiance of the plasma source is 210 mW/(mm^2^· sr · nm). The UV emission in the range of 300–370 nm emitted by the plasma is estimated to be 9 W, and the overall conversion efficiency from the total laser power to the mentioned UV emission power across 70 nm spectral range is ~10%. At this time, the laser power absorbed by the plasma is measured to be about 45 W. Hence, the calculated conversion efficiency of the plasma source from the absorbed laser power to the UV emission power (300–370 nm) is ~20%. To the best of our knowledge, we achieve a bright and compact UV-VIS-NIR source with the highest conversion efficiency. We then demonstrate its promising applications in spectral single-pixel imaging. Significant enhancement of 4 dB CNR in spectral single-pixel imaging has been demonstrated, compared with Xenon lamps of the same power consumption. Notably, this orthogonal LSP source shows the advantages of remarkable conversion efficiency, unprecedented brightness, compact structure, and low temporal coherence, which is essential to high-speed inspection and spectroscopy applications.

## Results

The low conversion efficiency of LSP is caused by the negative lensing effect of plasma. The negative lensing effect originates from the intrinsic refractive index distribution of plasma. The propagation process of electromagnetic waves in plasma will drive free electrons to generate current. According to Faraday’s law and Ampere’s law, the dispersion relationship of an electromagnetic wave of frequency *ω* and wave number *k* in plasma is given by ${\omega }^{2}-{c}^{2}{k}^{2}={{4\pi n}_{e}}^{2}/{m}_{e}$. Based on the definition formula of phase velocity^28^, the refractive index of the plasma is less than 1. According to the fluid model of plasma, the temperature at the LSP center is the highest, resulting in the highest electron density and the lowest refractive index. Therefore, the plasma can be approximated as a negative lens, causing the laser transmission path to deviate^29^. Under this negative lensing effect, laser beams are refracted and defocused, resulting in a decrease in laser power density^25,30^. Note that the conversion efficiency of LSP is positively correlated with the volume coefficient of the laser radiation absorption *μ* and the laser power density *I*_laser_ according to the energy conservation equation^31,32^. *μ* is related to the wavelength of the laser, the electron density *n*_*e*_ and the electron temperature *T*_*e*_ of the plasma. Raising gas pressure is a direct way to increase the electron temperature and the electron density^33,34^. Therefore, it is necessary to suppress the influence of the negative lensing effect on laser power density to achieve high-efficiency LSP.

**Fig. 1:**
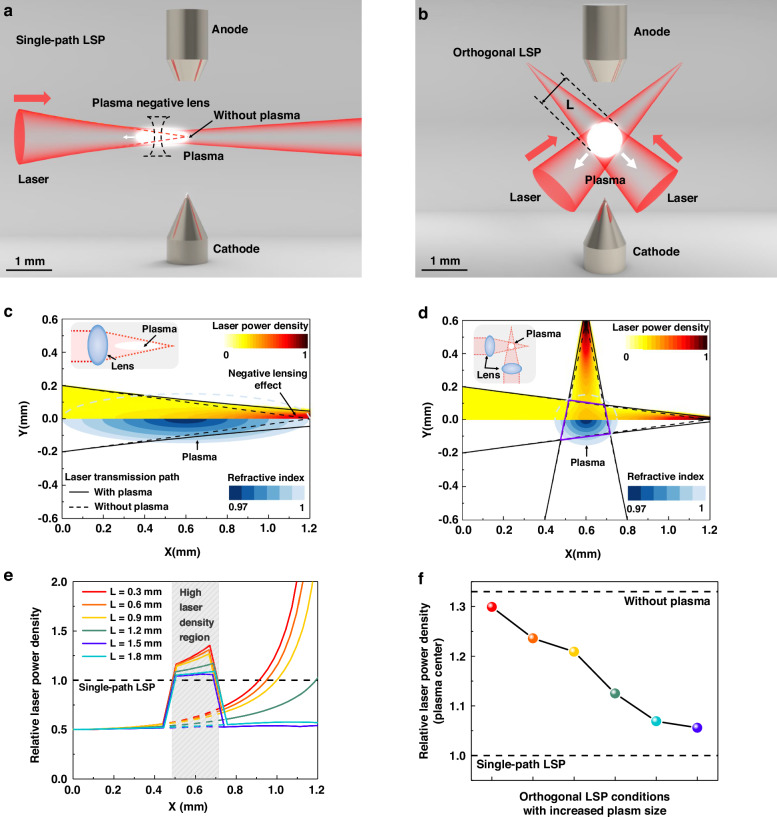
Simulation results of laser power density distribution. **a** Schematic diagram of single-path LSP. **b** Schematic diagram of orthogonal LSP. **c** Laser power density distribution of single-path LSP. **d** Laser power density distribution of orthogonal LSP. **e** Comparison of relative laser power density along *Y* = 0 axis. **f** Comparison of relative laser power density at plasma center (*X* = 0.6 mm, *Y* = 0)

The electron density in the central region of LSP is higher than that in the edge region, so the plasma center has the lowest refractive index. The spatial distribution of the refractive index in the plasma region can be equivalent to a plasma negative lens, which refracts and defocuses the incident laser. We establish a two-dimensional refractive index model in the plasma region to obtain the spatial distribution of laser power density. For conventional single-path LSP in Fig. 1a, the laser is incident from a single direction. Under the negative lensing effect, the laser transmission path is deflected by the plasma. Plasma grows in the direction of laser incidence, resulting in the shape of the plasma cross-section being close to an elliptical^35^. For orthogonal LSP in Fig. 1b, the shape of plasma cross-section gradually changes from circular, diamond-shaped to star-shaped with the increase of laser power^36^. We propose a hyperelliptic model to describe the two-dimensional refractive index spatial distribution of LSP with various shapes. We then calculate the laser transmission path in the plasma region based on the hyperelliptic model and the Eikonal equation^37^. Figure 1c demonstrates the spatial distribution of refractive index and laser power density corresponding to the single-path LSP. The refractive index is displayed by colored lines with the lowest value at the plasma center. Under the negative lensing effect, the laser transmission path deviates from the black dashed line to the solid line, and the laser power density decreases accordingly. Note that the laser is incident along the *Y* = 0 axis direction, so the plasma grows along the laser incidence direction. Therefore, the cross-sectional shape of the single-path LSP is elliptical with a high aspect ratio. In the case of orthogonal LSP, the laser is divided into two paths with the total laser power consistent with single-path LSP. As is shown in Fig. 1d, the deflection displacement of the laser transmission path is suppressed by ~75%. Meanwhile, the overlapping region of orthogonal lasers marked by purple lines has a higher laser power density. The increase in laser power density within the region will promote the growth in conversion efficiency. As the laser power further increases, the plasma will grow from circular to diamond-shaped and star-shaped. The color bar in the upper half of Fig. 1c, d represents the normalized laser power density distribution along the *X* axis direction. In the absence of plasma, the transmission path of the laser is shown by the dashed lines in Fig. 1c, d, with the same beam radius of 2.6 μm at the focal point (1.2, 0). When plasma is generated, it deflects the laser as a negative lens. For the single-path LSP in Fig. 1c, the plasma grows in the direction of laser incidence and has an approximately elliptical shape. For the orthogonal LSP in Fig. 1d, the shape is approximated to be circular with the orthogonal-path laser pumping together. In the presence of plasma, the transmission path of the laser is shown by the solid lines in Fig. 1c, d. The deviation of the laser transmission path in the single-path LSP is obvious, while the orthogonal design eliminates the influence of the negative lensing effect on laser power density.

We calculated six orthogonal LSP conditions with increased plasma size. The aspect ratio of the orthogonal LSP remains at 1, but the plasma length *L* increases from 0.3 mm to 1.8 mm. The relative laser power density distribution along the *Y* = 0 axis is shown in Fig. 1e. We set the relative laser power density of single-path LSP to 1. In the high laser density region marked by gray shadows, orthogonal lasers correspond to a higher power density than single-path LSP. As the plasma size increases, the suppression of laser transmission path deviation by orthogonal design will gradually decrease. The relative laser power density within the high laser density region will gradually decrease and approach the value corresponding to single-path LSP. Furthermore, the relative power density of the plasma center (*X* = 0.6 mm, *Y* = 0) is shown in Fig. 1f. When the plasma length is 0.3 mm, the interference of the negative lensing effect on laser power density is nearly eliminated. The above simulation results indicate that the orthogonal design redistributes laser power density in the spatial domain. The orthogonal design eliminates the negative lensing effect and constructs a high laser density region at relatively low laser power, thereby improving laser power density and promoting conversion efficiency. At relatively high laser power, the improvement of laser power density by orthogonal lasers will gradually weaken. In summary, based on the simulation results of higher laser power density, orthogonal LSP is expected to achieve higher conversion efficiency and brightness than single-path LSP at relatively low laser power.

**Fig. 2:**
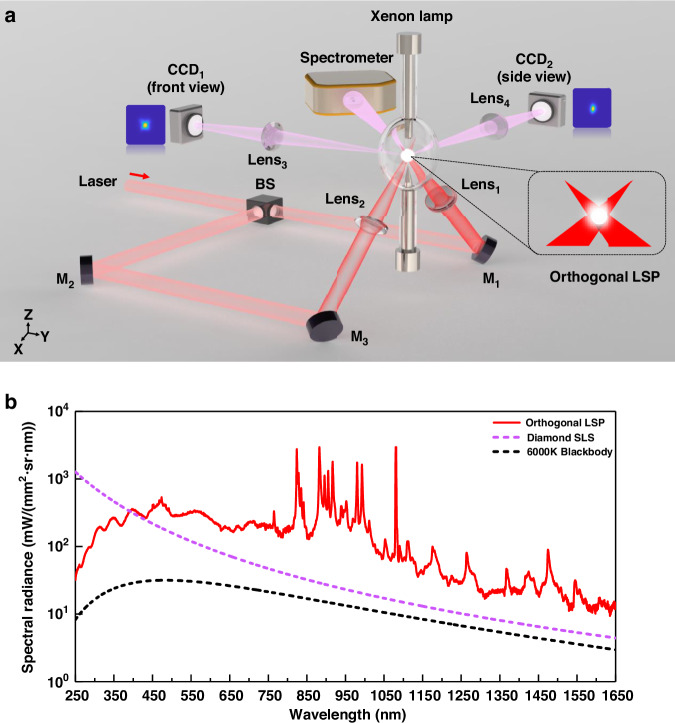
Experimental setup and spectral radiance of orthogonal LSP. **a** Schematic diagram of orthogonal LSP experimental set. **b** Comparison of spectral radiance among orthogonal LSP under 90 W laser power, Diamond synchrotron light source (SLS), and 6000 K Blackbody (surface temperature of the sun) in the wavelength range of 250–1650 nm

Based on theoretical analysis, an orthogonal LSP experimental setup is constructed, as shown in Fig. 2a. We provide a detailed introduction to the devices and measurement instruments in the “Materials and methods” section. A comparison of spectral radiance between orthogonal LSP and other UV-VIS-IR sources is shown in Fig. 2b. Spectral radiance of orthogonal LSP is significantly higher than that of Blackbody. According to our calculations, the continuous emission peak of orthogonal LSP corresponding to the current Xenon gas pressure is ~450 nm. Compared with the synchrotron light source, the difference in UV spectral radiance of 90 W orthogonal LSP is about one order of magnitude. This difference will be eliminated by increasing Xenon gas pressure to shift the peak wavelength of continuous emission to around 200 nm. We provide the calculation method for continuous emission intensity in the “Materials and methods” section.

**Fig. 3:**
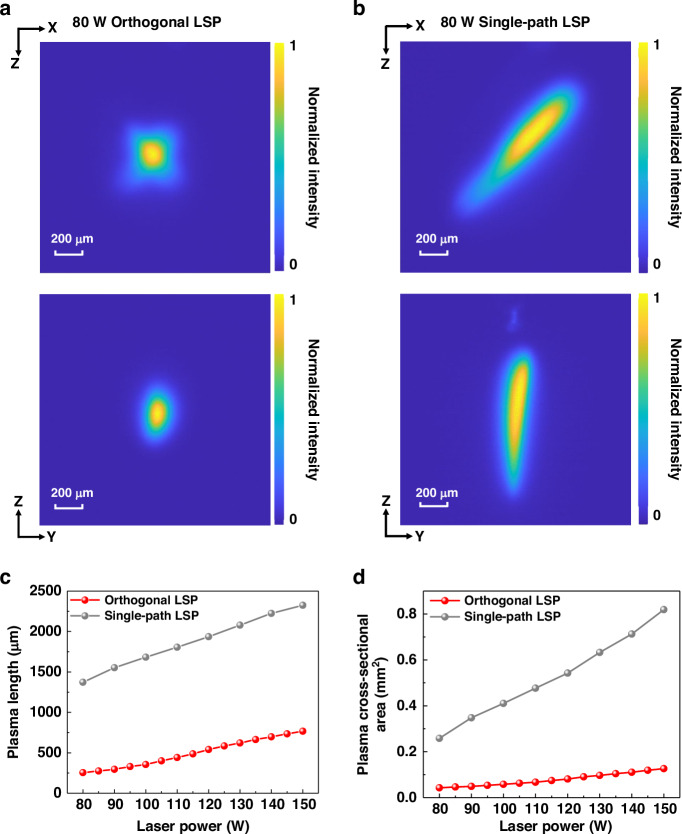
Measurement of LSP size. **a** Orthogonal LSP image in CCD_1_ (front view) and in CCD_2_ (side view). **b** Single-path LSP image in CCD_1_ (front view) and in CCD_2_ (side view). **c** Measurement results of plasma length. **d** Measurement results of plasma cross-sectional area in CCD_1_

The measurement of LSP size is shown in Fig. 3. CCD_1_ observes plasma at the front view (XOZ plane). CCD_2_ observes plasma at the side view (YOZ plane), proving that orthogonal lasers jointly sustain the same plasma. We compare the plasma image of single-path LSP and orthogonal LSP under the same total laser power. Single-path plasma has a higher aspect ratio and larger plasma size, which corresponds to the significant growth of plasma along the laser incidence direction caused by the negative lensing effect. On the contrary, orthogonal lasers suppress the size and aspect ratio growth of plasma. When laser power increases from 80 W to 140 W, the size of the orthogonal LSP increases, and the plasma changes from a symmetrical diamond-shaped to star-shaped. These CCD images of orthogonal LSP under different laser powers are shown in the Supplementary materials.

All images are normalized to obtain the plasma size. The full width at half maximum in the *Z* axis direction of CCD_2_ imaging is defined as the plasma length. Figure 3c shows the measurement results of plasma length in the laser power range of 80 W to 150 W. It can be observed that orthogonal lasers reduce the plasma length to about one-fifth of that of the single-path LSP and half the plasma length growth rate. The cross-sectional area of LSP is obtained by counting the pixels with intensity greater than half of the peak in CCD_1_ imaging. As is shown in Fig. 3d, orthogonal lasers significantly reduce the cross-sectional area of plasma and suppress its growth rate. The experimental results of plasma imaging demonstrate that orthogonal design can suppress the negative lensing effect. The laser power absorbed by the plasma will tend to increase the plasma temperature rather than just the plasma size, which is beneficial for improving the conversion efficiency of LSP.

**Fig. 4:**
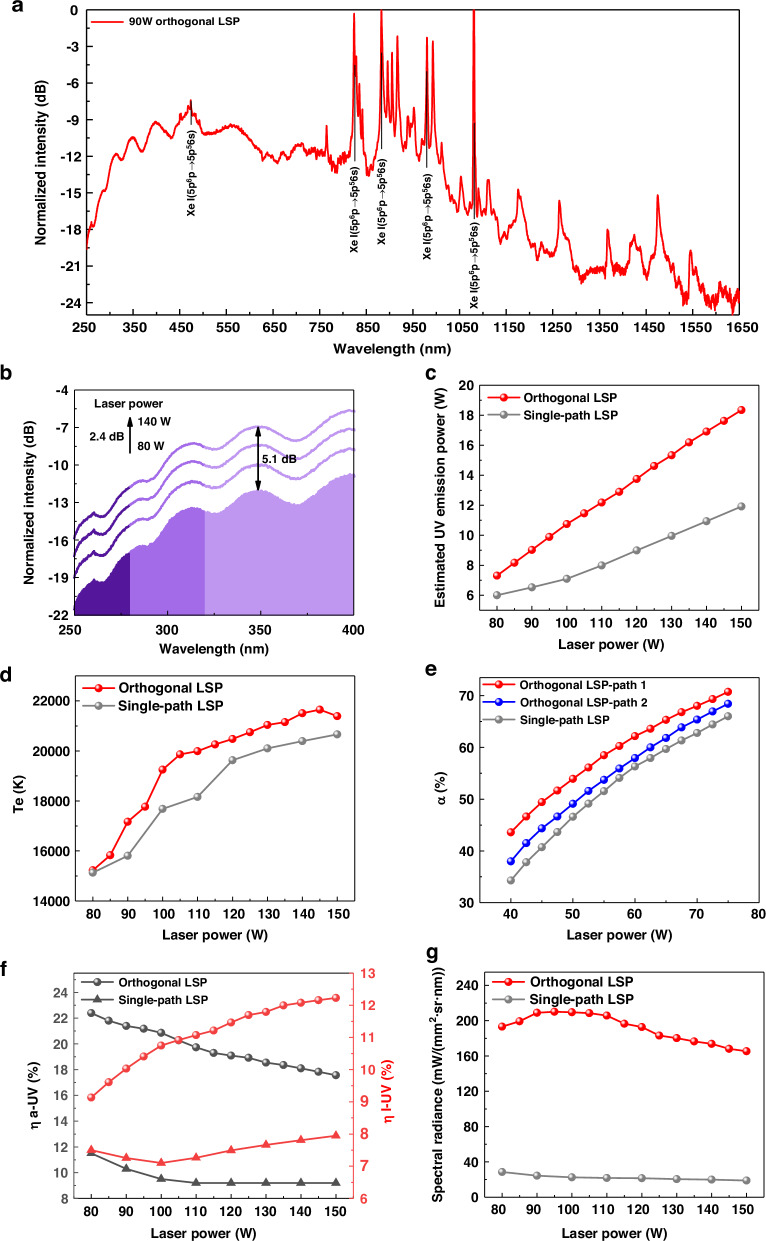
Conversion efficiency and brightness of the LSP source. **a** Normalized spectrum of orthogonal LSP and single-path LSP. **b** Variation of orthogonal LSP UV spectrum with laser power. **c** Estimated plasma UV emission power. **d** Diagnostic results of plasma electron temperature. **e** Measurement results of laser absorption rate by plasma. **f** Conversion efficiency from laser power absorbed by plasma to UV emission ${\eta }_{a-{UV}}$ and conversion efficiency from total incident laser power to UV emission ${\eta }_{l-{UV}}$. **g** Estimated plasma spectral radiance (300~370 nm)

The normalized spectrum of orthogonal LSP under 90 W laser is shown in Fig. 4a. LSP spectrum is composed of line emission and continuous emission. The typical line emission wavelength corresponding to the spontaneous emission process between the energy levels of Xenon atom is labeled. Figure 4b demonstrates that the 2.4 dB power increase of the orthogonal lasers resulted in a more than 5 dB spectral intensity increase. The difference in UV spectral intensity exceeds the difference in laser power, which corresponds to an improvement in conversion efficiency for orthogonal LSP. We compare the estimated plasma UV emission power, electron temperature, conversion efficiency, and spectral radiance of single-path LSP and orthogonal LSP under the same total laser power. In Fig. 4c, the estimated plasma UV emission power is calculated based on measurement results in the experiment. The spectral range for the measured UV emission power is 300–370 nm, which is the transmission band of the bandpass filter. The power meter is placed at a distance *R* from the plasma source center, and the bandpass filter is placed in front of the power meter. The radius of the power meter sensor is *r*, and the power measured by the power meter is *P*_*pm*_. Considering the nature of the Bremsstrahlung radiation for the LSP source, and *R* is much larger than the plasma length, so we assumed the plasma source emits uniformly into space within a 4π cubic angle. The UV emission power *P* within 300–370 nm can be calculated according to the following equation: $P=(4{R}^{2}/{r}^{2})\times {P}_{{\rm{pm}}}$. The results prove that the orthogonal design is beneficial for improving the UV emission power of the plasma and its growth rate with laser power. To analyze the reason for the increase in UV emission power, we use the line-to-continuum ratio method to diagnose the plasma electron temperature^38^. Based on the emission characteristics of Xenon plasma, we use the emission spectrum at 980 nm for the diagnosis. In Fig. 4d, we diagnose the electron temperature *t*_*e*_ of LSP based on the line-to-continuum ratio *R*_*lc*_ obtained in the spectral measurement and NIST database. The electron temperature of orthogonal LSP is ~1000 K higher than that of single-path LSP. The higher electron temperature leads to a higher laser power absorption rate by the plasma in Fig. 4e, thus increasing the laser power absorbed by plasma through reverse Bremsstrahlung. Note that the absorption rates of the plasma on the two branch lasers are different by ~5%, which might be due to the difference in the transmission path length of the two branch lasers within the plasma.

Subsequently, we describe the conversion efficiency and spectral radiance of the LSP source quantitatively in Fig. 4f, g. We define the conversion efficiency from total incident laser power to UV emission as ${\eta }_{l-{UV}}$ while the conversion efficiency from laser power absorbed by plasma to UV emission as ${\eta }_{a-{UV}}$. When the laser power is below 100 W, the conversion efficiency from laser power absorbed by plasma to UV emission of orthogonal LSP exceeds 20%, and the conversion efficiency from total incident laser power to UV emission exceeds 15%, significantly exceeding that of traditional single-path LSP. The high conversion efficiency enables bright UV sources with low power consumption. We estimate the average spectral radiance between 300 nm and 370 nm based on the method in our previous work. The estimation results indicate that the spectral radiance of orthogonal LSP is >7 times higher than that of single-path LSP. The average spectral radiance (300–370 nm) of orthogonal LSP is about 210 ${mW}/({{mm}}^{2}\,\cdot \,{sr}\,\cdot\, {nm})$ under 90 W laser. LSP source with the highest reported UV spectral radiance to our knowledge has been achieved.

The intrinsic characteristic of Bremsstrahlung and recombination emission makes the LSP source with low temporal coherence. We measure the spatial and temporal coherence of the orthogonal LSP source and Xenon lamp. The coherence measurement details are shown in the Supplementary materials^39^. The coherence area is used to describe and compare the spatial coherence of a light source. The coherence area of the orthogonal LSP is 0.401 mm^2^. We quantitatively characterize the temporal coherence by the coherence length, which is about 4 μm. The short coherence length indicates that orthogonal LSP source is suitable for imaging systems, which can avoid the influence of interference fringes and speckles on imaging results.

**Fig. 5:**
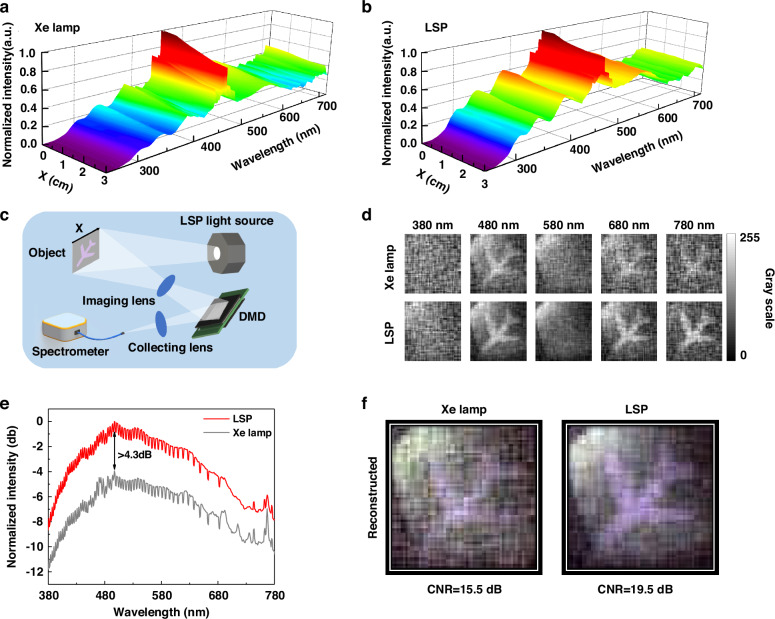
Application results of LSP source in spectral single-pixel imaging. **a** Spatial distribution of Xenon lamp spectral intensity on the object plane. **b** Spatial distribution of LSP source spectral intensity on the object plane. **c** Schematic diagram of spectral single-pixel imaging experimental setup. **d** Comparison of imaging results between LSP source and Xenon lamp at different wavelengths. **e** Comparison of normalized intensity measured by the spectrometer. **f** Comparison of reconstructed images and CNR calculation results

We use a spectrometer to compare the spatial intensity uniformity of the output light. Spectra at different positions from the optical axis within the object plane are plotted in Fig. 5a, b. For Xenon lamp, the spectral intensity will decrease by ~22.0% at a distance of 3 cm from the optical axis. For orthogonal LSP, the reduction in spectral intensity is ~9.5%. Based on the above advantages, we test the LSP source in spectral single-pixel imaging.

The experimental setup for spectral single-pixel imaging is shown in Fig. 5c. Broadband light illuminates the object, and the reflected light is imaged onto the digital micromirror device (DMD) through an achromatic lens. The beam modulated by DMD is finally focused into the spectrometer by a collection lens. The spectral resolution of the spectrometer is ~1 nm in the range of 380–780 nm. 64 × 64 pixel images with a sampling rate of 20% are obtained. The comparison of imaging results at different wavelengths is displayed in Fig. 5d. The purple pattern is printed with a mixture of blue and red ink, resulting in clearer imaging results at the corresponding wavelength. Figure 5e shows the comparison of normalized intensity measured by the spectrometer. The measurement results indicate that the high brightness characteristic of the LSP source increases the intensity of the signal detected by the spectrometer by up to 4.3 dB. The final imaging results of the object are reconstructed using data in the wavelength range of 380–780 nm, as shown in Fig. 5f. Significant enhancement of 4 dB CNR in spectral single-pixel imaging has been obtained, compared with Xenon lamp of the same power consumption.

## Discussion

In this paper, we establish a two-dimensional refractive index distribution in the plasma region to analyze the propagation path of the orthogonal lasers. The simulation results indicate that the orthogonal design eliminates the influence of the negative lensing effect on laser power density at relatively low laser power. When the laser power is 90 W, 70% of which is absorbed by the plasma, while the remaining 30% passes through the plasma and can be recycled by reflective optics. The use of air-cooled small-sized fiber lasers will significantly improve the compactness of the broadband source, ultimately achieving a broadband source that combines high brightness and miniaturization. In our case, the conversion efficiency from laser power absorbed by plasma to UV emission exceeds 20%, indicating a minimal thermal loss in such broadband source.

The average spectral radiance (300–370 nm) is about 210 ${mW}/({{mm}}^{2}\,\cdot\, {sr}\,\cdot\, {nm})$. We report an important conceptual advance that the orthogonal design eliminates the influence of the negative lensing effect on laser power density. Excitingly, under this conceptual advance, there is still potentials for improvement in the brightness of orthogonal LSP source. The essence of high brightness is to elevate the plasma density and temperature within a constraint region. The gas pressure inside the Xenon lamp is 1 MPa, we expect to further improve the brightness by using a higher-pressure chamber. Moreover, when laser power exceeds 90 W, the suppression of the negative lensing effect weakens with the increase in plasma size. With the plot of a two-dimensional refractive index model and the quantitative link between pumping optics design and plasma absorption, we believe that the negative lensing effect can be eliminated at higher laser power by dividing the laser into more beamlines and beam shaping.

We define a FOM (FOM = ∆λIP/V) to compare broadband sources comprehensively. *∆ λ* represents the spectral range of the broadband light source, *P* is the average power output by the light source, *I* is the brightness of the light source, and *V* is the footprint of the light source. The state-of-the-art for representative broadband sources are summarized in Table 1. The estimated FOM of orthogonal LSP is more than one order of magnitude higher than other broadband sources. Therefore, this work presents a compact broadband source that is essential to satisfy the demands in sensor calibration, metrology, microscopy, imaging, inspection, and spectroscopy applications.

## Materials and methods

### Analysis of laser transmission paths in the plasma region

We calculate the electron density by the Saha equation^30^:

1 $$\frac{{n}_{e}{n}_{i}}{{n}_{a}}=2{\left(\frac{2\pi mk}{h}\right)}^{3/2}\frac{{g}_{i}}{{g}_{a}}{{t}_{e}}^{3/2}\exp (-{E}_{i}/k{t}_{e})$$

where *n*_*e*_, *n*_*i*_, and *n*_*a*_ are the densities of electrons, ions, and neutral atoms, respectively. *h* is Planck’s constant, *k* is Boltzmann’s constant, *m* is the electron mass, *g*_*i*_ and *g*_*a*_ are statistical weights, *t*_*e*_ is the electron temperature, and *E*_*i*_ is the ionization potential of Xenon.

We then calculate the refractive index in the plasma region *n* as follows:

2 $$n=\sqrt{1-\frac{{\omega }_{p}^{2}}{{\omega }^{2}}}$$

where ${\omega }_{p}=\sqrt{\frac{4\pi {n}_{e}{e}^{2}}{m}}$ is the plasma frequency, *ω* is the laser frequency, and *e* is the electric charge. The refractive index at the plasma center in this work is ~0.97. Using a hyperelliptic model, we analyze the laser transmission path within the plasma region by the Eikonal equation^37^:

3 $$\left\{\begin{array}{c}\begin{array}{c}{\left(\frac{\partial u(x,y)}{\partial x}\right)}^{2}+{\left(\frac{\partial u(x,y)}{\partial y}\right)}^{2}={n}^{2}(x,y),(x,y)\in {{\mathrm{R}}}^{2}\\ u(x,y)=\varphi (x,y),(x,y)\in \varGamma \in {{\mathrm{R}}}^{2}\end{array}\\ {|\frac{x-{x}_{0}}{{h}_{1}}|}^{2a}+{|\frac{y-{y}_{0}}{{h}_{2}}|}^{2b}={|\frac{n(x,y)-0.97}{0.03}|}^{2c}\end{array}\right.$$

where *u*(*x*, *y*) is the laser propagation path. *φ*(*x*, *y*) is the boundary condition, *n*(*x*, *y*) is the refractive index of the plasma region and (*x*_0_, *y*_0_) is the coordinate of the plasma center. A, b, and c are the power exponentials that determine the two-dimensional refractive index distribution model.

### Experimental setup of orthogonal LSP

We divide the 1080 nm continuous laser into two beams by a 50/50 beam splitter. The beam diameter on both lenses is 10 mm, and the focal length of these lenses is 30 mm. The effective F-number is 3. The measured *M*^2^ of the laser is 1.25. The calculated beam radius at the focusing point is 2.6 μm, and the calculated Rayleigh range is 21 μm. Subsequently, the two laser beams pass through a series of planar mirrors and are focused by a pair of lenses in the orthogonal direction at the center of the Xenon lamp. For the Xenon lamp, the distance between the anode and the cathode is 2.5 mm, and the voltage applied to start the plasma is 10 kV. Plasma tends to move and grow along the direction of laser incidence driven by the electric field force. The focal points of orthogonal lasers are separated, so that a highly symmetrical plasma is sustained within the laser cross-region. Plasma emission comes from the region with a dimension of several hundred microns, which is much larger than the pumping beam dimensions at the focal point. The precision tuning of the off-focal crossing point maximizes the beam overlap and, hence, the plasma absorption. The plasma shape of the XOZ and YOZ cross-sections is measured through the lens and CCD imaging. The XOZ and YOZ cross-sections of LSP are defined as the front view and side view, respectively. We measure the plasma emission spectrum with a spectrometer, and we use a power meter to measure the plasma emission power within the corresponding solid angle. Finally, we calculate the spectral radiance based on the measurement results of plasma cross-sectional area, emission spectrum, and emission power.

### Electron temperature diagnosis method

The line emission intensity of the spontaneous transition from upper-level *u* to lower-level *l* satisfies the following formula^38^:

4 $${\varepsilon }_{l}=\frac{{A}_{ul}hc}{4\pi {\lambda }_{ul}}\frac{{n}_{e}{n}_{i}{g}_{u}}{2{U}_{i}(T{t}_{e})}\frac{{h}^{3}}{(2\pi mk{t}_{e})}\exp (\frac{{E}_{i}-{E}_{u}}{k{t}_{e}})$$

where *c* is the speed of light, *A*_*ul*_ is the Einstein emission coefficient, *g*_*u*_ is the statistical weight of high-level *u*, *λ*_*ul*_ is the central wavelength, *U*_*i*_ is the partition function of xenon ions, *E*_*i*_ is the Rydberg energy, and *E*_*u*_ is the energy of the upper-level *u*. Continuous emission comes from recombination and Bremsstrahlung processes in which free electrons participate in LSP. The continuous emission coefficient *I*_*c*_ is approximately constant within a limited spectral bandwidth range *Δλ*. Therefore, the relationship between continuous emission intensity *ε*_*c*_ and continuous emission coefficient *I*_*c*_ is ${\varepsilon }_{c}={I}_{c}\varDelta \lambda$. *I*_*c*_ can be approximated by the following formula:

5 $${I}_{c}={C}_{1}\left(\frac{{n}_{e}{n}_{i}}{{{\lambda }_{ul}}^{2}\sqrt{{t}_{e}}}\right)\xi ({\lambda }_{ul},{t}_{e})$$

where the constant ${C}_{1}=\frac{16\pi {e}^{6}}{3{c}^{2}\sqrt{6\pi {m}^{3}k}}$, $\xi \left({\lambda }_{{ul}},{t}_{e}\right)$ is the Bibermann correction factor at the central wavelength *λ*_*ul*_ and temperature *t*_*e*_. The line-to-continuum ratio *R*_*lc*_ is a function of the LSP electron temperature:

6 $${R}_{lc}=\frac{{\varepsilon }_{l}}{{I}_{c}\varDelta \lambda }={C}_{2}\frac{{A}_{ul}{g}_{u}}{{t}_{e}{U}_{i}\xi ({\lambda }_{ul},{t}_{e})}\frac{{\lambda }_{ul}}{\varDelta \lambda }\exp \left(\frac{{E}_{i}-{E}_{u}}{k{t}_{e}}\right)$$

where the constant ${C}_{2}=\frac{{3}^{3/2}{h}^{4}{c}^{3}}{256{\pi }^{3}{e}^{6}k}$. We diagnose the electron temperature *t*_*e*_ of LSP based on the line-to-continuum ratio *R*_*lc*_ obtained in the spectral measurement and NIST database.

### Spectral single-pixel imaging

Single-pixel imaging is based on second-order correlation of light, which has the advantage of high sensitivity^40–42^. Spectral single-pixel imaging is a method that combines high spectral and spatial resolution^43,44^. The light reflected from the object is modulated by DMD and collected into the spectrometer. The signal intensity detected by the spectrometer can be expressed as follows:

7 $${S}_{ij}=\iint I(x,y,{\lambda }_{j}){P}_{i}(x,y)dxdy$$

where *x* and *y* correspond to the two-dimensional spatial information of the object and *λ* corresponds to the wavelength information of the object. *I* is the spatial intensity distribution of the original image. *S*_*ij*_ corresponds to the detection ordinal number *i* and the wavelength ordinal number is *j*. *P* is the spatial distribution of the modulated speckle pattern loaded by DMD. In this experiment, DMD is loaded with 1639 matrix patterns and switched sequentially at a refresh rate of 8 Hz. The integration time of the spectrometer is 100 ms to ensure that the collected signal strength is sufficient for image reconstruction. For a selected wavelength, we reconstruct the grayscale imaging result from the signal collected by the spectrometer and speckle patterns loaded in DMD. We subsequently reconstruct the colorful imaging of the object using data in the 380–780 nm range. We use CNR for the quantitative comparison of image quality.

8 $$CNR=10{\log }_{10}\left(\frac{|{\mu }_{o}-{\mu }_{b}|}{\sqrt{{\sigma }_{o}^{2}+{\sigma }_{b}^{2}}}\right)$$

*μ*_*o*_ and *μ*_*b*_ are the average gray value of object and background, while *σ*_*o*_ and *σ*_*b*_ are the standard deviation of the gray value of object and background.

## Supplementary information

Supplementary Material for Bright Compact Ultrabroadband Source by Orthogonal Laser-sustained Plasma

## References

[1] Munkhbat, B. et al. Tunable self-assembled Casimir microcavities and polaritons. *Nature* **597**, 214–219 (2021). 34497392 10.1038/s41586-021-03826-3

[2] Standing, A. et al. Efficient water reduction with gallium phosphide nanowires. *Nat. Commun.* **6**, 7824 (2015). 26183949 10.1038/ncomms8824 PMC4518318

[3] Hong, C. Y. et al. Engineering electrode interfaces for telecom-band photodetection in MoS_2_/Au heterostructures via sub-band light absorption. *Light Sci. Appl.* **12**, 280 (2023). 37996413 10.1038/s41377-023-01308-x PMC10667329

[4] Ding, N. et al. A novel approach for designing efficient broadband photodetectors expanding from deep ultraviolet to near infrared. *Light Sci. Appl.* **11**, 91 (2022). 35410451 10.1038/s41377-022-00777-w PMC9001727

[5] Pi, L. J. et al. Broadband convolutional processing using band-alignment-tunable heterostructures. *Nat. Electron.* **5**, 248–254 (2022).

[6] Ward, M. B. et al. Superoxide dismutase activity enabled by a redox-active ligand rather than metal. *Nat. Chem.* **10**, 1207–1212 (2018). 30275506 10.1038/s41557-018-0137-1

[7] Aldag, P. et al. Dynamic interplay between target search and recognition for a Type I CRISPR-Cas system. *Nat. Commun.* **14**, 3654 (2023). 37339984 10.1038/s41467-023-38790-1 PMC10281945

[8] Chen, X. Q. et al. Imaging the transient heat generation of individual nanostructures with a mechanoresponsive polymer. *Nat. Commun.* **8**, 1498 (2017). 29138401 10.1038/s41467-017-01614-0 PMC5686141

[9] Wagner, M. et al. Ultrabroadband nanospectroscopy with a laser-driven plasma source. *ACS Photonics* **5**, 1467–1475 (2018).

[10] Shi, N. N. et al. Nanostructured fibers as a versatile photonic platform: radiative cooling and waveguiding through transverse Anderson localization. *Light Sci. Appl.* **7**, 37 (2018). 30839604 10.1038/s41377-018-0033-x PMC6107007

[11] Hentschel, M. et al. Dielectric Mie voids: confining light in air. *Light Sci. Appl.* **12**, 3 (2023). 36587036 10.1038/s41377-022-01015-z PMC9805462

[12] Shrestha, S. et al. Room temperature valley polarization via spin selective charge transfer. *Nat. Commun.* **14**, 5234 (2023). 37633986 10.1038/s41467-023-40967-7 PMC10460417

[13] Kaza, N., Ojaghi, A. & Robles, F. E. Ultraviolet hyperspectral microscopy using chromatic-aberration-based iterative phase recovery. *Opt. Lett.* **45**, 2708–2711 (2020). 32412447 10.1364/OL.392634

[14] Liu, Z. W. et al. Flexible hyperspectral surface plasmon resonance microscopy. *Nat. Commun.* **13**, 6475 (2022). 36309515 10.1038/s41467-022-34196-7 PMC9617892

[15] Zhu, J. L. et al. Optical wafer defect inspection at the 10 nm technology node and beyond. *Int. J. Extrem. Manuf.* **4**, 032001 (2022).

[16] Bechtel, H. A. et al. Ultrabroadband infrared nanospectroscopic imaging. *Proc. Natl Acad. Sci. USA* **111**, 7191–7196 (2014). 24803431 10.1073/pnas.1400502111 PMC4034206

[17] Deng, X. J. et al. Experimental demonstration of the mechanism of steady-state microbunching. *Nature* **590**, 576–579 (2021). 33627811 10.1038/s41586-021-03203-0

[18] Jiang, X. et al. Deep-ultraviolet to mid-infrared supercontinuum generated in solid-core ZBLAN photonic crystal fibre. *Nat. Photonics* **9**, 133–139 (2015).

[19] Sollapur, R. et al. Resonance-enhanced multi-octave supercontinuum generation in antiresonant hollow-core fibers. *Light Sci. Appl.* **6**, e17124 (2017). 30167225 10.1038/lsa.2017.124 PMC6062021

[20] Elu, U. et al. Seven-octave high-brightness and carrier-envelope-phase-stable light source. *Nat. Photonics* **15**, 277–280 (2021).

[21] Hong, L. H. et al. Intense ultraviolet-visible-infrared full-spectrum laser. *Light Sci. Appl.* **12**, 199 (2023). 37607910 10.1038/s41377-023-01256-6 PMC10444876

[22] Jin, S. L. et al. Compact ultrabroadband light-emitting diodes based on lanthanide-doped lead-free double perovskites. *Light Sci. Appl.* **11**, 52 (2022). 35256583 10.1038/s41377-022-00739-2 PMC8901751

[23] Zhang, H. Y. et al. Single-crystal perovskite light-emitting diodes with external quantum efficiency of over 8% enabled by nonstoichiometric composition tuning. *Laser Photonics Rev.* **17**, 2200904 (2023).

[24] Wu, J. H. et al. Near-infrared laser driven white light continuum generation: materials, photophysical behaviours and applications. *Chem. Soc. Rev.* **49**, 3461–3483 (2020). 32338256 10.1039/c9cs00646j

[25] Shi, Z. J. et al. Investigation of the laser-sustained plasma of a Xenon lamp driven by an annular beam. *Opt. Express* **31**, 6132–6142 (2023). 36823877 10.1364/OE.480954

[26] Horne, S. et al. A novel high-brightness broadband light-source technology from the VUV to the IR. *Proceedings of SPIE 7680, next-generation spectroscopic technologies III*. Orlando: SPIE, 76800L (2010).

[27] Bezel, I. et al. High power laser-sustained plasma light sources for KLA-Tencor broadband inspection tools. *Proceedings of CLEO: applications and technology 2015*. San Jose: Optica Publishing Group (2015).

[28] Hur, M. S. et al. Laser pulse compression by a density gradient plasma for exawatt to zettawatt lasers. *Nat. Photonics* **17**, 1074–1079 (2023).

[29] Zimakov, V. P. et al. Spatial and temporal instabilities of optical discharges. *Fluid Dyn.* **57**, S84–S96 (2022).

[30] Yang, S. C. et al. Laser-sustained plasma of high radiance in the ultraviolet spectral range based on the reservoir effect of the annular beam. *Opt. Express* **31**, 25625–25634 (2023). 37710444 10.1364/OE.496045

[31] Rafatov, I. Effect of focusing geometry on the continuous optical discharge properties. *Phys. Lett. A* **373**, 3336–3341 (2009).

[32] Liu, J. B., Zhang, D. H. Y. & Fu, Y. Y. Formation and evolution of multiple-core structures in laser-sustained plasmas. *N. J. Phys.* **25**, 122001 (2023).

[33] Murphy, A. B. & Tam, E. Thermodynamic properties and transport coefficients of arc lamp plasmas: argon, krypton and xenon. *J. Phys. D: Appl. Phys.* **47**, 295202 (2014).

[34] Kotov, M. A. et al. Oscillations of convective flow around a continuous optical discharge in high-pressure Xenon. *Plasma Sources Sci. Technol.* **31**, 124002 (2022).

[35] Hu, Y. F., Wang, X. B. & Zuo, D. L. Research of continuous fiber laser sustained Xe plasma. *Vacuum* **203**, 111229 (2022).

[36] Kulumbaev, É. B. & Lelevkin, V. M. Continuous optical discharge in crossed laser beams. *Plasma Phys. Rep.* **26**, 617–620 (2000).

[37] Kulyabov, D. S. et al. Numerical analysis of eikonal equation. *Proceedings of SPIE 11066*, Saratov Fall Meeting 2018: laser physics, photonic technologies, and molecular modeling. Saratov: SPIE, 2018, 110660U (2018).

[38] Lu, Q. et al. Characteristic diagnostics of a laser-stabilized high-pressure argon plasma by optical emission spectroscopy. *IEEE Trans. Plasma Sci.* **50**, 2578–2587 (2022).

[39] Born, M. & Wolf, E. *Principles of Optics*. 7th edn. (Cambridge: Cambridge University Press, 1999).

[40] Wang, F. et al. Far-field super-resolution ghost imaging with a deep neural network constraint. *Light Sci. Appl.* **11**, 1 (2022). 34974515 10.1038/s41377-021-00680-w PMC8720314

[41] Wang, Y. Q. et al. Mid-infrared single-pixel imaging at the single-photon level. *Nat. Commun.* **14**, 1073 (2023). 36841860 10.1038/s41467-023-36815-3 PMC9968282

[42] Xiong, J. H. et al. Perovskite single-pixel detector for dual-color metasurface imaging recognition in complex environment. *Light Sci. Appl.* **12**, 286 (2023). 38008796 10.1038/s41377-023-01311-2 PMC10679139

[43] Li, W. L. et al. Dual-color terahertz spatial light modulator for single-pixel imaging. *Light Sci. Appl.* **11**, 191 (2022). 35739086 10.1038/s41377-022-00879-5 PMC9225988

[44] Martins, G. B. et al. OpenSpyrit: an ecosystem for open single-pixel hyperspectral imaging. *Opt. Express* **31**, 15599–15614 (2023). 37157658 10.1364/OE.483937

