# Supplementary material for: Bright compact ultrabroadband source by orthogonal laser-sustained plasma
Source: Light Sci Appl. 2024 Sep 26;13:274. doi: 10.1038/s41377-024-01602-2 (PMC11427449; doi:10.1038/s41377-024-01602-2)
Supplement: Supplementary file 1 — Supplementary Material for Bright Compact Ultrabroadband Source by Orthogonal Laser-sustained Plasma [file 41377_2024_1602_MOESM1_ESM.docx]

**Supplementary Material for Bright Compact Ultrabroadband Source by Orthogonal Laser-sustained Plasma**

Zhaojiang Shi,^1^ Shichao Yang,^1^ He Hu, ^1^ Haodong Lei, ^1^ Zhaohua Yang, ^1^ and Xia Yu ^1,🖂^

Correspondence: Xia Yu (xiayu@buaa.edu.cn)

^1^School of Instrumentation and Optoelectronic Engineering, Beihang University, Beijing 100191, China

These authors contributed equally: Zhaojiang Shi, Shichao Yang

1. **CCD images of orthogonal LSP under different laser power**

**
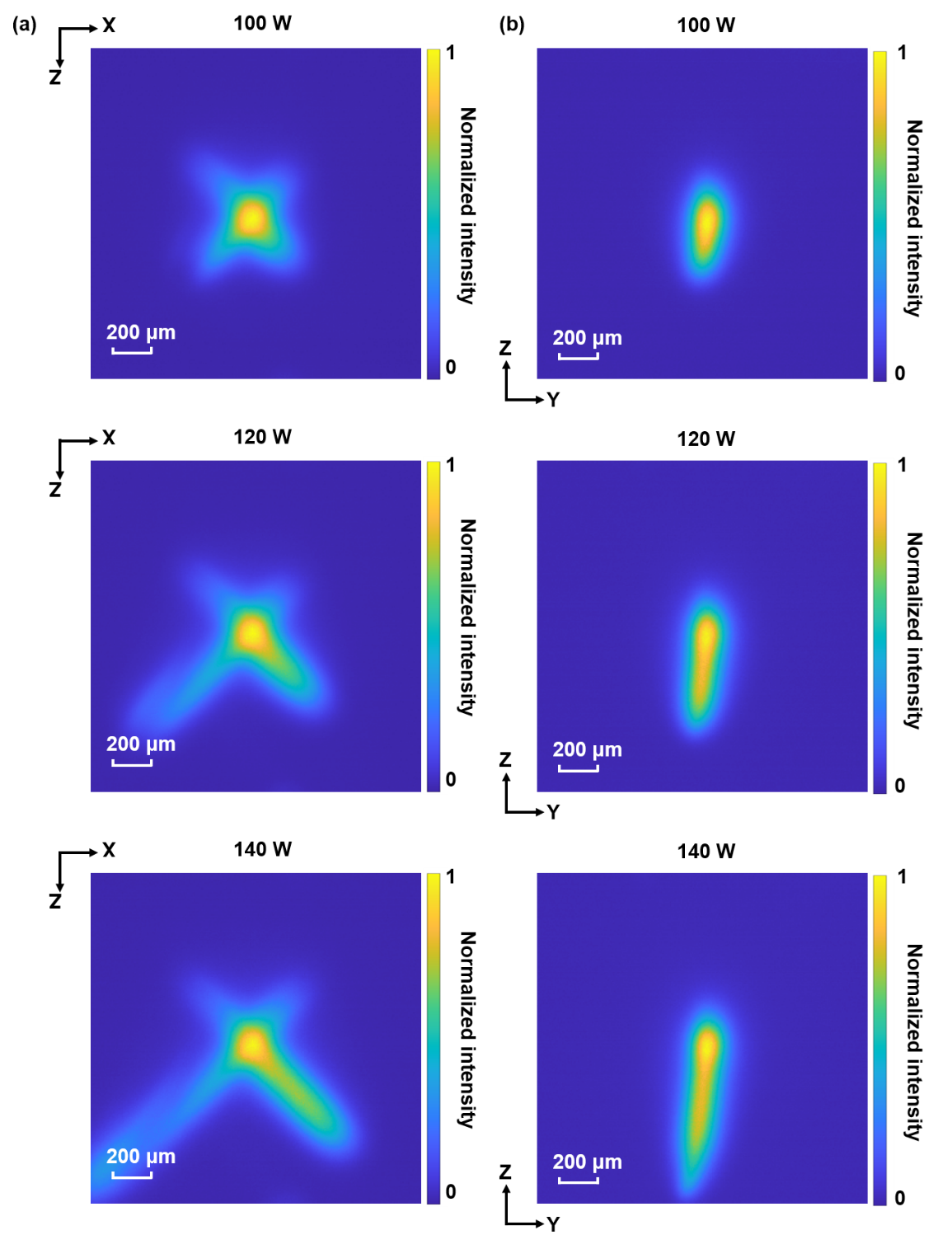
**

**Fig. S1: CCD images of orthogonal LSP under different laser power** (a) Orthogonal LSP image in CCD (front view). (b) Orthogonal LSP image in CCD (side view).

1. **Measurement for coherence**
2. **The spatial coherence of light source**

According to the van Cittert–Zernike theorem, even for incoherent light sources, there may be a certain degree of coherence between the two points $P_{1}$ and $P_{2}$ in its radiation field as shown in the following Fig. S2.


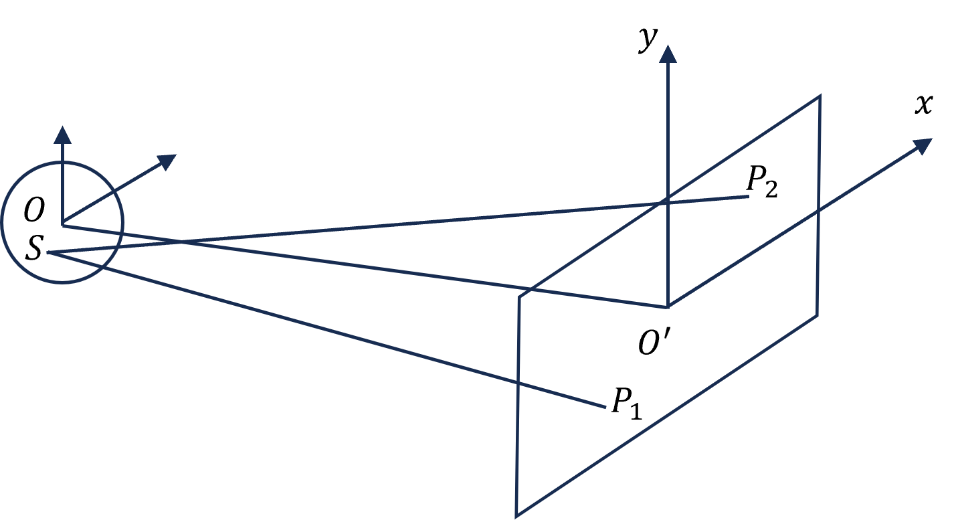


**Fig. S2: Schematic diagram of van Cittert–Zernike theorem**

The complex coherence $j_{12}$ between $P_{1}\left( X_{1}, Y_{1} \right)$and $P_{2}(X_{2},Y_{2})$ can be expressed as (*Born M, Wolf E. Principles of Optics,7^th^ ed*):

$$j_{12}=(\frac{2J_{1}(v)}{v})e^{i\psi}$$

where $J_{1}$ is the first type of first-order Bessel function, $v=\frac{2\pi}{\bar{\lambda}}\frac{\rho}{R}d$and $\psi=\frac{2\pi}{\bar{\lambda}}\left[ \frac{\left( X_{1}^{2}+Y_{1}^{2} \right)-(X_{2}^{2}+Y_{2}^{2})}{2R} \right]$. $\bar{\lambda}$ is the mean wavelength of the light source. $R$is the distance $OO^{'}$from the light source to the $O^{'}P_{1}P_{2}$ plane. $\rho$ is the radius of the light source. $(X_{1},Y_{1})$, $\left( X_{2},Y_{2} \right)$ are the coordinates of $P_{1}$and $P_{2}$ referred to parallel axes at $O^{'}.$ $d=\sqrt{{(X_{1}-X_{2})}^{2}+{(Y_{1}-Y_{2})}^{2}}$ is the distance between $P_{1}$and $P_{2}$. $\psi$ represents a phase difference of$2\pi(OP_{1}-OP_{2})/ \bar{\lambda}$. It can be ignored when $OP_{1}-OP_{2}\ll\bar{\lambda}$ in Young's double-slit experiment (d=100$\mu m$, R=0.3 m). At this point, the coherence $\left| j_{12} \right|$ equals to:

$$\left| j_{12} \right|=\left| \frac{2J_{1}(v)}{v} \right|$$

In our experiment, we set $R=0.3 m$, $\bar{\lambda}=525 nm$, radius of LSP $\rho_{LSP}=134.5 \mu m$, radius of arc plasma in Xenon lamp $\rho_{Xe}=250 \mu m$. The relationship between the coherence$\left| j_{12} \right|$of the light source (LSP source and Xenon lamp) and the distance $d$ can be calculated as follows:


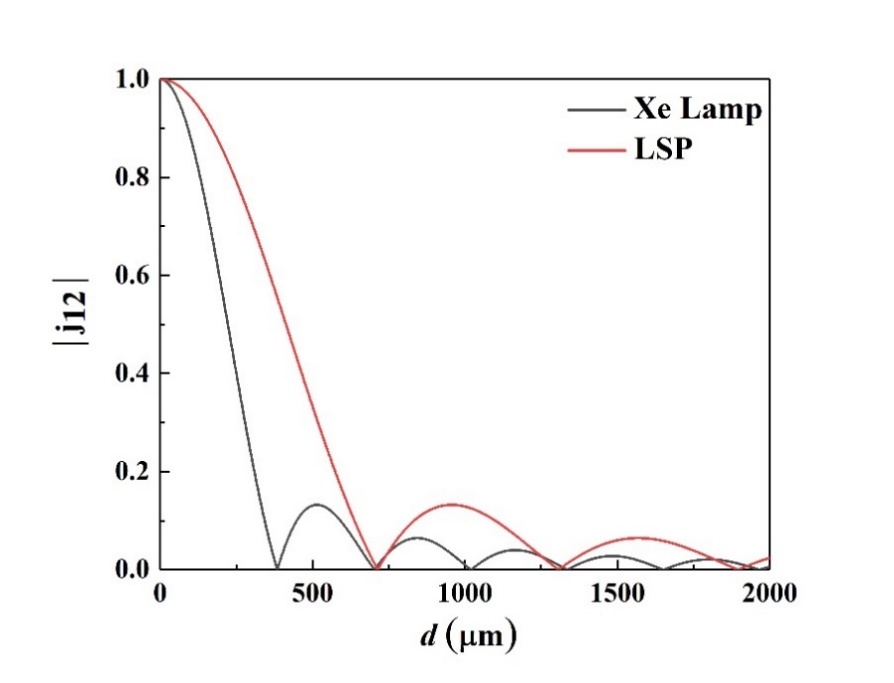


**Fig. S3:** **The calculated coherence of LSP source and Xenon lamp**

The coherence area is usually used to describe and compare the spatial coherence of a light source. It can be found that the coherence of the Xenon lamp drops to 0 for the first time when $d=384.1 \mu m$. When $d=714.3 \mu m$, the coherence of the LSP source drops to 0 for the first time. At a distance of $0.3 m$ from the Xenon lamp, there exists a coherence region with a diameter of $d=384.1 \mu m$ and a coherence area$S$ $=\frac{\pi}{4}d^{2}=0.116 {mm}^{2}$. At a distance of $0.3 m$ from the LSP light source, there exists a coherence region with a diameter of $d=714.3 \mu m$ and a coherence area $S=0.401 {mm}^{2}$.

To verify the above calculations, we conducted the following experiments. The experimental setup is shown in Fig. S4 (a). A bandpass filter with a central wavelength of 525 nm and the transmission bandwidth of 80 nm is placed after the light source to reduce the effect of non-monochromaticity on the visibility of the interference fringes. A double-slit with a spacing of $d=100 \mu m$ is placed at a distance $R=0.3 m$ from the light source. The slit widths equal to 30 μm. The interference fringes of the LSP source obtained by CCD is shown in Fig. S4 (b). The intensity distribution of the interference fringes along the X-axis is shown in Fig. S4 (c), where $I_{max}$ and $I_{min}$ are the maximum and minimum intensity of zero order interference fringe, respectively. Note that the noise floor caused by environmental stray light is deducted during the data processing.


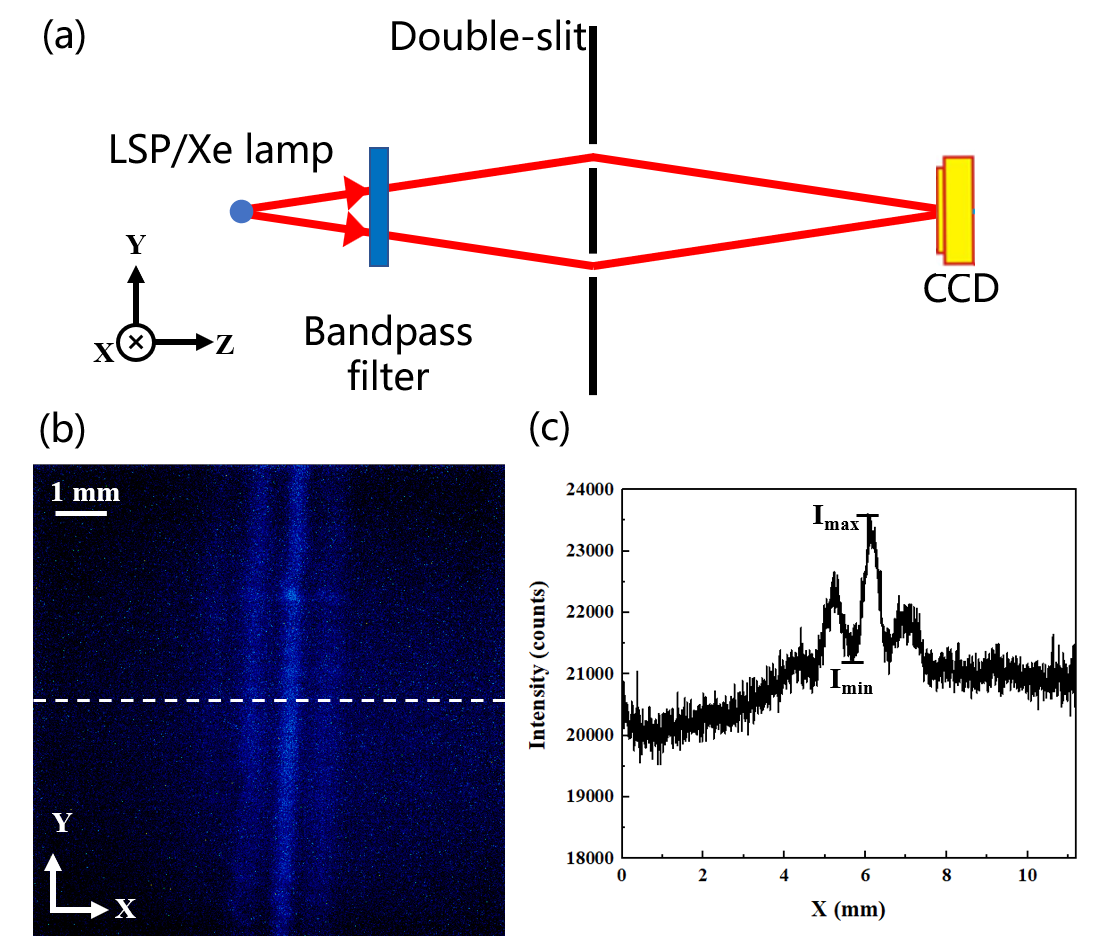


**Fig. S4:** **Spatial coherence measurement based on double-slit experiment.** (a) Schematic diagram of experimental setup. (b) The interference fringes of the LSP source obtained by CCD. (c) The intensity distribution of the interference fringes along the X-axis.

The visibility $V$ of the interference fringes can be calculated by

$V=\frac{I_{max}-I_{min}}{I_{max}+I_{min}}=\frac{2\sqrt{I_{1}}\sqrt{I_{2}}}{I_{1}+I_{2}}\left| j_{12} \right|$.

For this experiment, we have carried three sets of measurement using double-slit with different separation $d$, including 100 μm, 200 μm and 400 μm. all the slit widths equal to 30 $\mu m$, so there is $I_{1}=I_{2}$, i.e., $V=\left| j_{12} \right|$. The experimental results fit well with the theoretical calculations, as shown in Fig. S5. The black dots in the figure show the visibility of the interference fringes of the Xenon lamp under double-slit with different spacings $d$, and the red dots show the visibility of the interference fringes of the LSP source. The solid lines are the coherence of the Xenon lamp and the LSP source calculated according to the van Cittert-Zernike theorem.


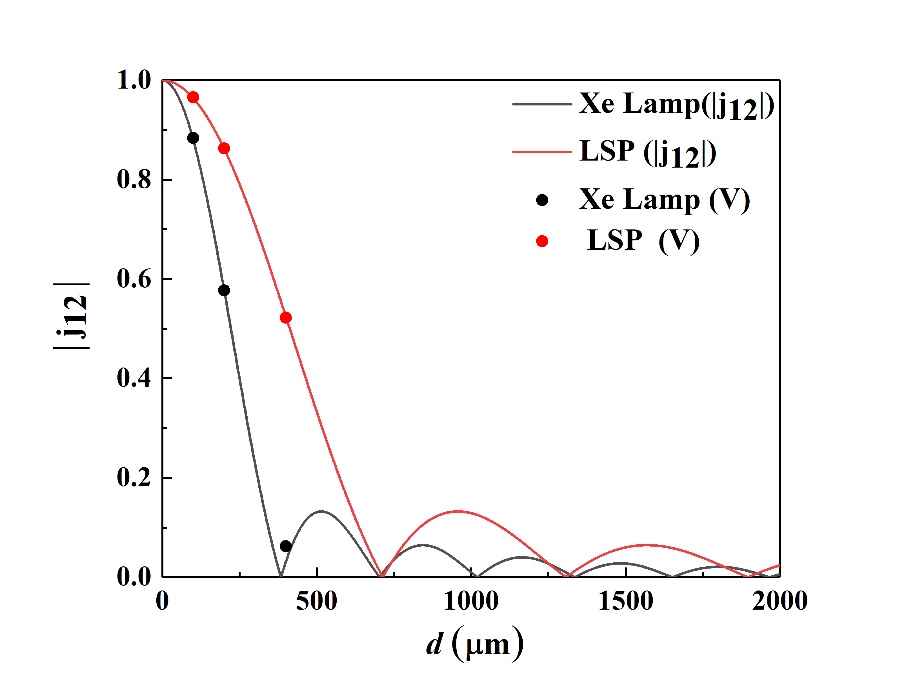


**Fig. S5: The calculated and measured coherence of LSP source and Xenon lamp**

1. **The temporal coherence of light source**

We have added the schematic diagram and moved all the details of temporal coherence measurement to the supplementary materials.

The output light of the LSP source is collimated by a lens with a focal length of 100 mm, and divided into two beams by a 50/50 non-polarized beam splitter. Two beams are reflected by a pair of planar mirrors $M_{1}$ and $M_{2}$ and pass through the beam splitter, ultimately interfering on the observation plane at a small angle. We adjust the two arms of the interferometer to have approximately equal optical path lengths to observe interference fringes on CCD. We place one of the planar mirrors of the interferometer on a displacement stage with a resolution of 1 μm. During the translation process of the planar mirror, interference fringes obtained by CCD will appear in the center of the observation plane and then disappear. The displacement of the planar mirror corresponding to the appearance and disappearance of interference fringes is the coherence length of the light source.

The coherence length of the LSP source was measured to be 4 μm. After replacing the source with a Xenon lamp, the coherence length was still 4 μm. Therefore, the temporal coherence of the LSP source is approximately the same as that of the Xenon lamp.


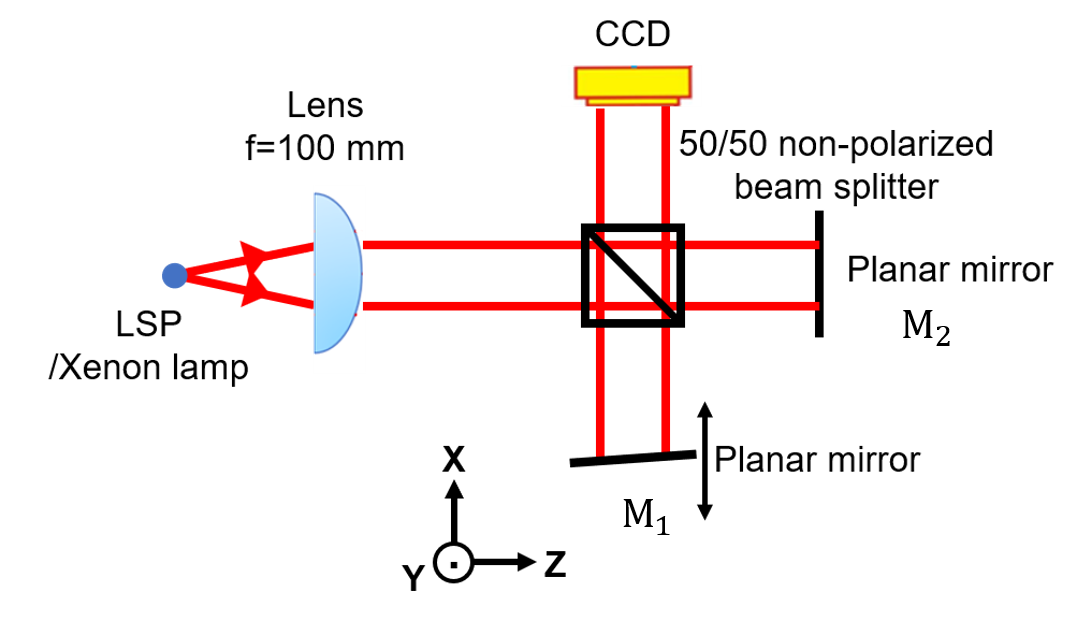


**Fig. S6: Schematic diagram of temporal coherence measurement based on Michelson interferometer**
